# Cross-Species Surveillance of Respiratory Viruses in Domestic and Wild Mammals of an Urban Atlantic Forest from Brazil

**DOI:** 10.1007/s10393-024-01691-w

**Published:** 2025-02-04

**Authors:** Leonardo Corrêa da Silva Junior, Deborah Fernandes Wailante, Marina Galvao Bueno, Patricia Emilia Bento Moura, Alex Pauvolid-Corrêa, Roberto Leonan Morim Novaes, Sócrates Fraga da Costa-Neto, Iuri Veríssimo, Natasha Avila Bertocchi, Ricardo Moratelli, Rosana Gentile, Fernando Couto Motta, Mia Ferreira de Araújo, David Brown, Paola Cristina Resende, Marilda Agudo Mendonça Teixeira de Siqueira, Maria Ogrzewalska

**Affiliations:** 1 Laboratório de Vírus Respiratórios, Exantemáticos, Enterovírus e Emergências Virais, Fundação Oswaldo Cruz, IOC, Rio de Janeiro, RJ 21040-900 Brazil; 2 Laboratório de Virologia Comparada e Ambiental, Fundação Oswaldo Cruz, IOC, Rio de Janeiro, RJ 21040-900 Brazil; 3 Laboratório de Virologia Veterinária de Viçosa, Departamento de Veterinária, Universidade Federal de Viçosa, Viçosa, MG 36570-900 Brazil; 4 Fundação Oswaldo Cruz, Fiocruz Mata Atlântica, Rio de Janeiro, RJ 22713-570 Brazil; 5 Laboratório de Biologia e Parasitologia de Mamíferos Silvestres Reservatórios, Fundação Oswaldo Cruz, IOC, Rio de Janeiro, RJ 21040-360 Brazil

**Keywords:** Zoonosis, Respiratory viruses, One health, Wildlife, South America

## Abstract

Our aim was to investigate respiratory viruses circulating in animals from the urban Atlantic Forest, which is located in the most densely populated area near Rio de Janeiro in Brazil. This study focused on the detection of Influenza A viruses and diverse coronaviruses, including SARS-CoV-2, in domestic and wild animals, including bats, nonhuman primates, rodents, and marsupials. From August 2020 to September 2022, biological samples were collected from a total of 72 pets, 66 primates, 20 rodents, 36 marsupials, and 390 bats. Samples were tested using RT-PCR for Influenza A and coronaviruses, and positive samples were sequenced. When blood samples were available, they were tested for SARS-CoV-2 antibodies. All terrestrial animals were negative for evidence of infection with SARS-CoV-2 and Influenza A viruses. However, samples from 17 phyllostomid bats, including Great fruit-eating bat (*Artibeus lituratus*), Silky short-tailed bat (*Carollia brevicauda*), Seba's short-tailed bat (*Carollia perspicillata*), Common big-eared bat (*Micronycteris microtis*), Greater spear-nosed bat (*Phyllostomus hastatus*), White-lined broad-nosed bat (*Platyrrhinus lineatus*), Little yellow-shouldered bat (*Sturnira lilium*), Greater round-eared bat (*Tonatia bidens*), and Common vampire bat (*Desmodus rotundus*), were positive for bat coronaviruses classified as *Alphacoronavirus*. Our study adds new information on the occurrence of coronaviruses in bats and contributes to a long-term program of Influenza surveillance. Developing active surveillance for viruses in wildlife species, as implemented in this study, is crucial for understanding zoonotic risks and preventing future global pandemics.

**Supplementary Information:**

The online version contains supplementary material available at 10.1007/s10393-024-01691-w.

## Introduction

The increasing scale of unsustainable occupation of neotropical habitats is widely recognized as a risk for exposing humans to viruses hosted by wildlife (Daszak et al. 2000; Cunningham et al. 2017; Gibb et al. 2020). Human population growth and associated changes, including rising resource demands, act as a catalyst for environmental transformations such as urbanization, agricultural expansion, and habitat alteration. These factors play a pivotal role in the emergence and reemergence of infectious diseases by influencing ecological systems at the landscape and community levels, as well as by changing the dynamics of host and pathogen populations (Jones et al. 2008, 2013).

The emergence of viral diseases has had a significant impact on public health throughout history, with more than 70% of these diseases traced back to wildlife origins (Jones et al. 2008). Specifically, RNA viruses originating from wildlife hosts have been responsible for the emergence of many impactful diseases in humans, including influenza A, Marburg and Ebola fever, severe acute respiratory syndrome (SARS), Middle East respiratory syndrome (MERS), and more recently, coronavirus disease (COVID-19) (Woolhouse et al. 2005; Parrish et al. 2008; Zaki et al. 2012; Zhou et al. 2020). The occurrence of these and a range of other human infections resulted from a viral agent spilling over from wildlife hosts to humans, and subsequently spreading within human populations (Parrish et al. 2008). To protect future public health, it is crucial to identify amplifying hosts for such viruses, to establish and conduct surveillance to detect host-jumping events, and to comprehend the viral and host factors that may facilitate these occurrences (Dobson et al. 2020; Gibb et al. 2020).

Originally spanning an area of over 1.1 million km^2^ across 17 Brazilian states, the Atlantic Forest is globally recognized as a biodiversity hotspot (Mittermeier et al. 2000). It is the most threatened biome in Brazil, facing challenges from habitat fragmentation and loss. The biome is currently reduced to less than 25% of its original coverage (Vancine et al. 2024), and major Brazilian cities, such as Rio de Janeiro with a population of 6.7 million inhabitants, are situated within it. This phenomenon not only endangers the biome itself but also brings people and domestic animals into closer contact with wild animals and intensifying the risk of zoonotic transmission.

Coronaviruses (Coronaviridae, CoVs) contain important mammalian pathogens, including SARS-CoV-2 (Cui et al. 2019). Infection with SARS-CoV-2, which causes COVID-19, is a human illness that likely originated from wildlife and escalated into a global pandemic due to extensive human-to-human transmission. This novel zoonotic virus is characterized by its ability to infect both humans and certain animal species, with spillover and spillback events occurring through close contact between animals and humans (Zhang et al. 2021; Prince et al. 2021; Yen et al. 2022).

SARS-CoV-2 was confirmed to have rapidly spread to every federative unit of Brazil by May 30th, 2020 (MS 2020) and the establishment of SARS-CoV-2 transmission in the Brazilian population raised concerns not only about the imminent catastrophic impact on the population but also regarding the potential spillback of SARS-CoV-2 to wildlife and domestic animals, as seen elsewhere around the world (Cibulski et al. 2020; Olival et al. 2020; Colunga-Salas and Hernandez-Canchola 2021). Fears regarding SARS-CoV-2 infection in wildlife included the potential for sustained transmission in one or more Brazilian mammalian species, resulting in novel reservoirs of SARS-CoV-2. Other concerns include the potential for evolution in these species leading to increased virulence or recombination with other enzootic viruses, generating novel entities with the potential to cause disease in wildlife, domestic animals, and humans (Olival et al. 2020).

Influenza A viruses are among the most significant zoonotic pathogens. These viruses have been identified in a wide range of mammals and are particularly prevalent in wild aquatic birds, which are considered the natural reservoirs for avian influenza viruses (AIVs) (Webster et al. 1992). The pandemics of influenza, such as the Spanish influenza of 1918–1919, the 1957 Asian influenza, the 1968 Hong Kong influenza, the 1977 Russian influenza, and the 2009 swine flu pandemic, highlight the profound impact that viral host-switching can have on public health and the global economy (Horimoto and Kawaoka 2005; Trifonov et al. 2009). The host range of influenza viruses has been extensively studied to understand the role of various animals in the transmission chain—animals once thought unlikely to be involved in the spread of the virus. However, there remains a significant gap in our knowledge regarding the prevalence and impact of influenza in wild animals in Brazil.

Thus, our objectives in this study were to (i) investigate pathogens in wildlife addressing the research gap in Brazil by systematically studying pathogens in wild animals, particularly focusing on coronaviruses (including SARS-CoV-2) and respiratory viruses like Influenza A, (ii) evaluate viral genetic diversity of viruses found in various hosts and reservoirs within the Atlantic Forest reserve near Rio de Janeiro, and (iii) explore the interconnectedness of viral circulation among wildlife, domestic animals, and human populations to better understand the transmission dynamics and potential spillover events. Our main hypotheses were that (i) wild animals in the Atlantic Forest act as reservoirs for diverse and potentially zoonotic viruses, including coronaviruses and Influenza A viruses, and (ii) there is a potential for cross-species transmission of viruses between wildlife, domestic animals, and humans in the densely populated areas surrounding the urban Atlantic Forest.

## Methods

### Study Area

**Figure 1:**
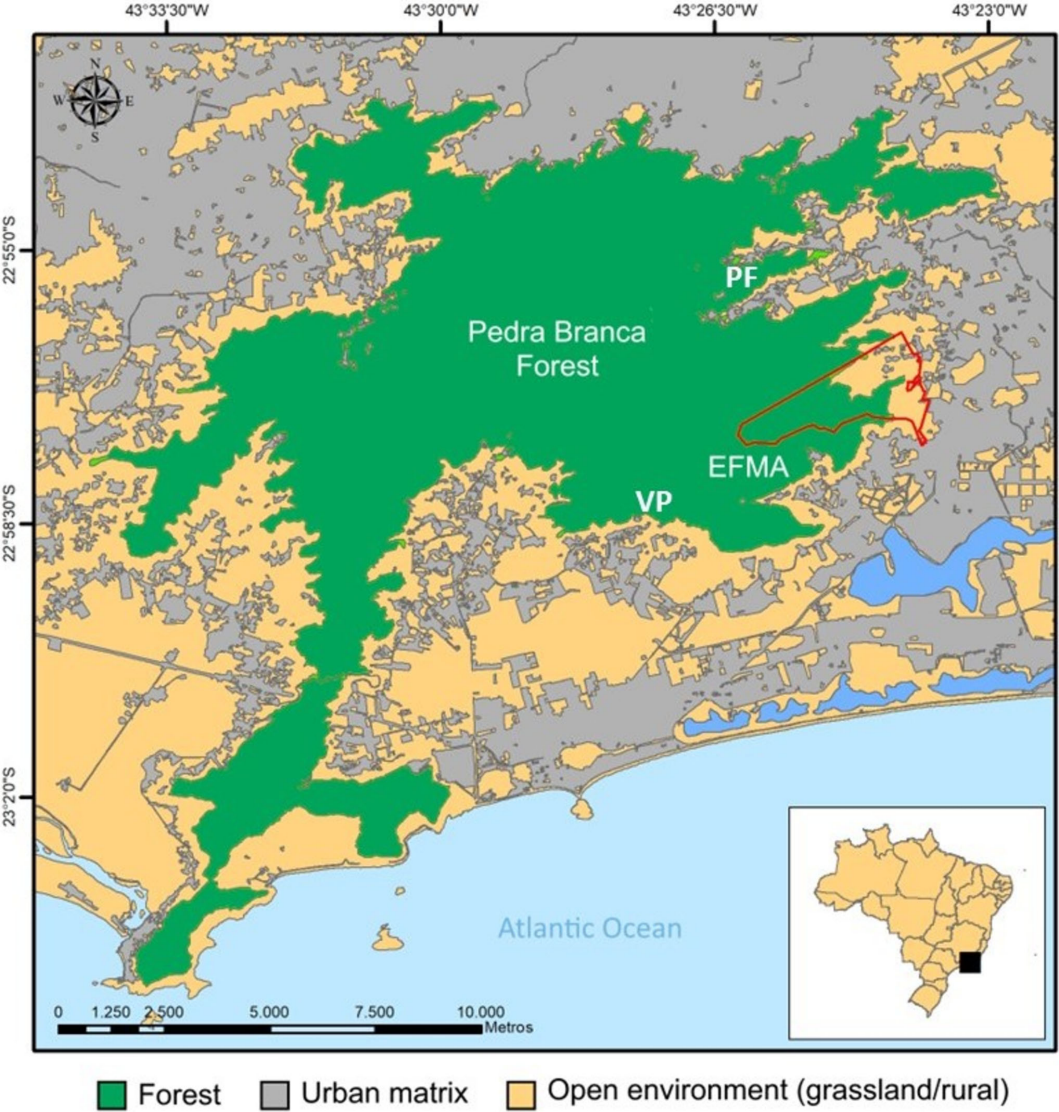
Location of the Fiocruz Atlantic Forest Biological Station (EFMA; central coordinates 22°56′25" S, 43°24′18″W, red line) in the context of the remaining Pedra Branca Forest (green) and urbanized matrix (gray) at Rio de Janeiro city, Southeastern Brazil. VP—Vargem Pequena locality (22º97′38.06″ S, 43º45′47.26’’ W), PF—Pau da Fome locality (22°93′31.31″ S, 43°44′10.5″ W). (Source: Generated by authors using QGIS based on public governmental shapefiles) (Color figure online).

**Figure 2:**
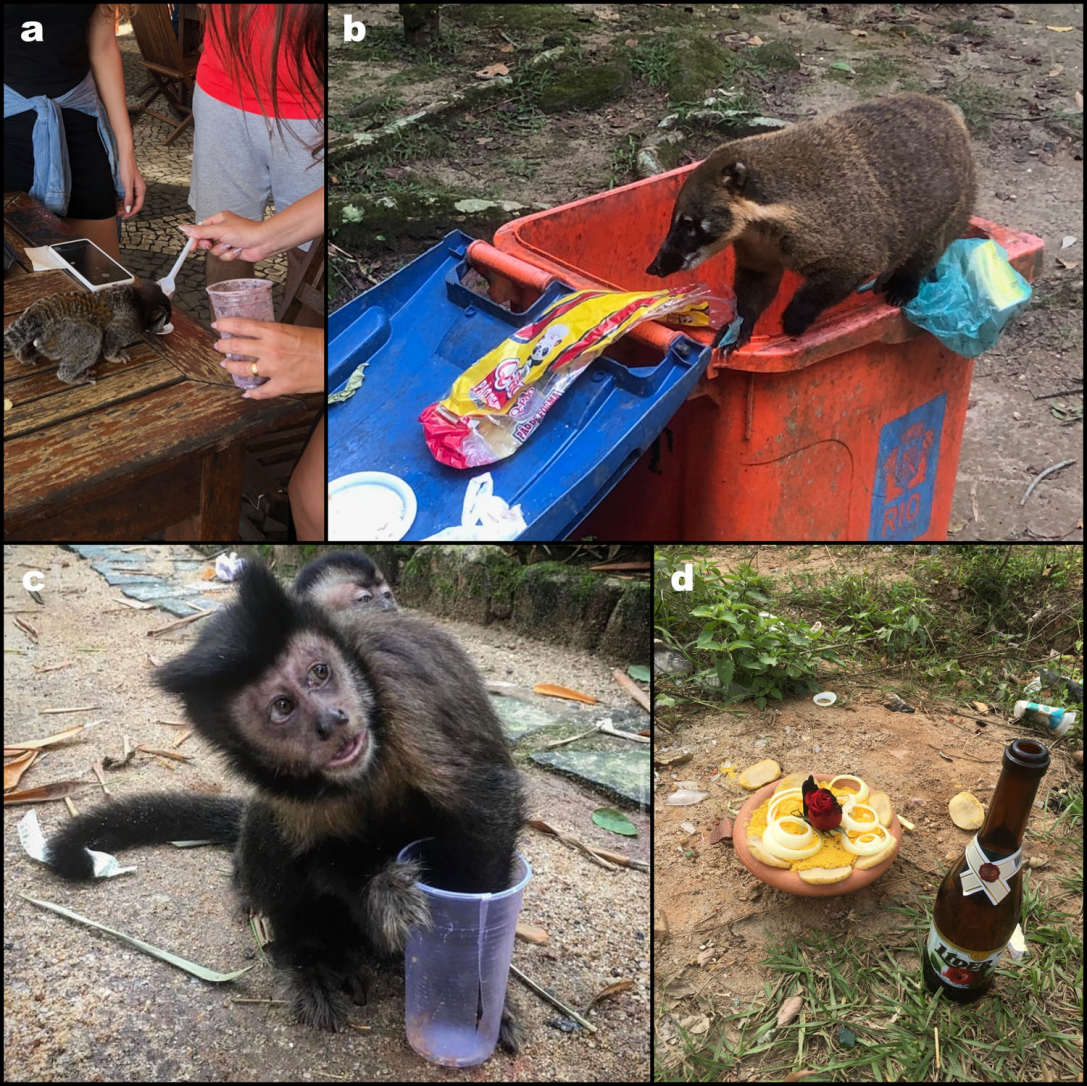
Interaction of wildlife with humans within the Atlantic rainforest in Rio de Janeiro city. A—Marmoset (*Callithrix* sp.) fed by children; B—South American coati (*Nasua nasua* (Linnaeus, 1766)) looking for food in trash; C—Capuchin monkeys (*Sapajus nigritus* (Goldfuss, 1809)) eating from trash; D—food left for religious reasons, attracting wildlife. Image credit: Maria Ogrzewalska.

The study was conducted in areas of the Fiocruz Atlantic Forest Biological Station (EFMA; 462 ha; 22°56′18″S, 43°24′11″W), and in areas of the Pedra Branca State Park (PEPB) in the localities of Vargem Pequena (22°93′31″S, 43°44′10″W) and Pau da Fome (22º97′38″S, 43º45′47’’W), municipality of Rio de Janeiro, Brazil (Fig. 1). These localities are within the Pedra Branca Forest (PBF), the largest urban forest in the world, covering an area of more than 13,000 ha (Moratelli et al. 2023). The Pedra Branca Forest consists of a mosaic of landscapes, incorporating preserved forest, areas in intermediate and initial stages of reforestation, and peridomicile zones with consolidated urbanization. The lowland areas within the peridomicile are characterized by high social vulnerability and precarious sanitation conditions. These areas are interconnected at various levels with densely populated neighborhoods. Alongside a high demographic occupation, the area experiences additional anthropogenic impacts, including small-scale agricultural and poaching activities (Moratelli et al. 2023). On the border with the forest, wild animals interact with domestic animals and humans, and they are frequently seen looking for food in human settlements and trash (Fig. 2). Samplings of all wild animals were conducted in areas adjacent to human occupancy to focus on those animals with a higher level of interaction with humans.

### Legal and Ethical Issues

The project was authorized by ICMBio (Chico Mendes Institute for Biodiversity Conservation, 19,037–1, 13,373–1), Environmental Institute of Rio de Janeiro State (INEA020/2011), and National System for the Management of Genetic Heritage and Associated Traditional Knowledge (SisGen AFAC9D5, AAB9E36, A46B0E1). All procedures followed the guidelines of the Ethical Committee on Animal Use of the Oswaldo Cruz Foundation (CEUA/Fiocruz LM6-18, L-36/18, LW57-19, LW58-19).

### Animal Sampling

Domestic cats and dogs from local squatter settlements were sampled between August 2020 and May 2022. After physical restraint, blood samples (3–5 mL) were collected through cephalic or jugular vein venipuncture. Respiratory samples for each animal comprised one oropharyngeal swab (rayon, 3–4 mm thick) and two nasal swabs (2 mm thick, Flexible Minitip Flocked Swab, FLOQSwabs®) all placed together in a single sterile tube containing viral transport medium (VTM). Fecal samples consisted of a single swab (Rayon, 3–4 mm thick) inserted into the anal sphincter and immediately placed into tubes containing VTM. The samples were kept refrigerated for up to 4 h before being frozen at -80 °C.

Invasive species marmosets (hybrid of Common marmoset *Callithrix jacchus* (Linnaeus, 1758) and Black-tufted marmoset *Callithrix penicillata* (Geoffroy 1812)) were sampled from July 2020 to August 2022 using tomahawk traps installed on suspense platforms attached to trees. After capturing the entire group of marmosets, each individual was subjected to an anesthesia protocol for the collection of biological samples. Given that these animals are exotic invaders, euthanasia is recommended as a population control measure. Consequently, a comprehensive set of samples was collected to support both this study and future research initiatives. Blood samples (8–12 mL) were obtained via cardiac puncture following anesthesia. Additionally, oropharyngeal and rectal swabs were collected as previously described.

Small mammal captures were carried out from September 2021 to September 2022 in five sampling campaigns. Three campaigns were carried out in EFMA during four consecutive nights in peridomicile and disturbed forest areas. One campaign was carried out in Vargem Pequena and one in Pau da Fome during eight consecutive nights each, all in preserved forests. Mammals were collected using Tomahawk® (16 × 5 × 5 inches) and Sherman® (3 × 3.75 × 12 inches) live-traps, both suitable for the capture of live animals up to 3 kg. Four linear transects of 20 points spaced in 20 m were established in each area. Two traps were placed on the ground at each point. All traps were baited with a mixture of peanut butter, banana, oatmeal, and bacon. The total trapping effort was 640 trap-nights in EFMA and 1280 trap-nights in Vargem Pequena and Pau da Fome. Marsupials and rodents (except for lactating marsupials) were transported to a field laboratory base for euthanasia and sample collection procedures. The animals were anesthetized and submitted to euthanasia for blood and tissue collection, according to recommended safety procedures and under the Guidelines for the Care and Use of Laboratory Animals, Oswaldo Cruz Foundation, Brazil (FIOCRUZ, License number L-36/2028). The blood samples (3–5 mL) were collected through cardiac puncture, after anesthesia procedure. Oral and rectal swabs were collected and preserved as described for above. Marsupials were identified by external and cranial morphology. Rodents were identified by external morphology, cranial morphology, and cytogenetic (chromosome counting—2N and FN—Corrêa et al., 2023) and molecular (gene sequencing) analyses, when necessary. All identifications were coordinated by the Reference Laboratory for Taxonomy of Reservoir Wild Mammals which is part of the Laboratory of Biology and Parasitology of Reservoir Wild Mammals of the Oswaldo Cruz Institute (Andrea et al., 2021). The mammalian specimens were submitted to taxidermy and deposited in the Integrated Collection of Wild Reservoir Mammals (COLMASTO) of the Oswaldo Cruz Institute.

Bats were sampled from October 2020 to September 2022 using ground-level mist nets. At the field laboratory base, the captured animals were identified and had their bionomic data collected and submitted to oral and rectal swab sampling as well as feces sampling, when available. Bats were not submitted to euthanasia, thus no blood samples were collected.

### Virus Detection

Swabs were submitted directly to RNA extraction, while fecal samples were first clarified as described before (Ogrzewalska et al. 2022). Extracted RNA was tested for SARS-CoV-2 and Influenza A using a commercial kit Biomanguinhos designed to amplify the envelope protein (E) gene of SARS-CoV-2 according to (Corman et al. 2020) using E_Sarbeco_F 5’-ACAGGTACGTTAATAGTTAATAGCGT, E_Sarbeco_R, 5’-ATATTGCAGCAGTACGCACACA-3’ and probe E_Sarbeco_P1 FAM-ACACTAGCCATCCTTACTGCGCTTCG-BBQ, and all influenza A types using the following primers for matrix gene (M) F-5’GACCRATCCTGTCACCTCTGAC-3’, R 5’-AGGGCATTYTGGACAAAKCGTCTA-3’, probe 5’-TGCAGTCCTCGCTCACTGGGCACG-3 according to (Shu et al. 2011). Reactions were conducted in a Veriti Thermo Cycler (Applied Biosystems) with the following conditions: reverse transcription (50°C, 15 min), reverse transcriptase inactivation, and DNA polymerase activation (95°C, 2 min), followed by 40 cycles of DNA denaturation (94°C, 20 s), annealing (58°C, 30 s). All the samples were also tested in a pancoronavirus RT-PCR targeting the RNA-dependent RNA polymerase (*RdRp*) gene as described previously (Chu et al. 2011). In brief, cDNA was synthesized and amplified in a first-round PCR using the One-Step RT-PCR Enzyme Mix Kit (Qiagen). The primers used in the first round were RdRp S1 (5´-GGKTGGGAYTAYCCKAARTG-3') and RdRp R1 (5'-TGYTGTSWRCARAAYTCRTG-3'), generating an expected product size of 602 base pairs (bp). Reactions were conducted in a Veriti Thermo Cycler (Applied Biosystems) with the following conditions: reverse transcription (50°C, 30 min), reverse transcriptase inactivation, and DNA polymerase activation (95°C, 15 min), followed by 40 cycles of DNA denaturation (94°C, 45 s), annealing (52°C, 45 s), and extension (72°C, 45 s), concluding with a final extension step (72°C, 10 min). Subsequently, nested PCR was carried out using the Phusion RT-PCR Enzyme Mix kit (Sigma-Aldrich), along with primers Bat1F (5’-GGTTGGGACTATCCTAAGTGTGA-3’) and Bat1R (5’-CCATCATCAGATAGAATCATCAT-3’). One microliter of the amplified product from the first round was used as a template in the nested PCR, conducted under the following conditions: denaturation (98°C, 30 s), followed by 35 cycles of DNA denaturation (98°C, 15 s), annealing (52°C, 15 s), extension (72°C, 30 s), and a final extension step (72°C, 5 min). The RdRp amplicons, approximately 440 bp in size, were visualized on 1.5% agarose gels using SYBR™ Safe DNA Gel Stain (Thermo Fisher Scientific). The positive controls utilized for PCR consisted of RNA samples of SARS-CoV-2 extracted from isolated virus, following the same procedures outlined for animal samples. The Sanger sequencing reaction was prepared using BigDye Terminator v3.1 Cycle Sequencing Kit (Life Technologies) using the ABI 3730 DNA Analyzer (Applied Biosystems) (Otto et al. 2008).

### Phylogenetic Analyses

The reads generated by Sanger sequencing were evaluated and assembled using Sequencher 5.1 (GeneCodes). The obtained consensus was checked by chromatogram analysis, and the final consensus was uploaded to the National Center for Biotechnology Information (NCBI) GenBank database. For CoV species and type assignment, nucleotide sequences were analyzed by the Basic Local Alignment Search Tool (BLAST) available in NCBI (https://blast.ncbi.nlm.nih.gov/Blast.cgi).

The *RdRp* gene partial sequences were used to construct a dataset for phylogenetic analyses (Supplementary Table 1) and were compiled to include (i) sequences obtained in this work (n = 8); (ii) sequences of American bat *Alphacoronavirus* previously identified belonging to clades A to G by (Caraballo 2022) (n = 129); (iii) other *Alphacoronavirus* sequences from the American continent available in GenBank on October 2023 (n = 71); as outgroup genus *Betacoronavirus* sequences (n = 12). The sequences were aligned with MAFFT v.7 (Katoh and Standley 2013), using default parameters. Bayesian phylogenetic analysis was performed with MrBayes v.3.2.7 (Ronquist et al. 2012) using the GTR + I + G model with gamma distribution and invariant sites, through Cipres Computational Resources (Miller et al. 2010). The Markov Chain Monte Carlo (MCMC) was run for 100,000,000 iterations, with trees saved every 100,000 iterations, after a 2,500 burn-in.

### ***Plaque Reduction Neutralization Test (PRNT***_***90***_***)***

A PRNT_90_ was used to detect neutralizing antibodies to SARS-CoV-2 (Okba et al. 2020; Calvet et al. 2021). The infectious B.1 variant SARS-CoV-2 (EPI_ISL_414045) used was isolated from a human patient from Rio de Janeiro, Brazil. Briefly, the samples were initially screened at a dilution of 1:10 and those that neutralized virus challenge by at least 90% in Vero CCL-81 cells were further tested at serial twofold dilutions that ranged from 1:10 to 1:320 to determine 90% reduction endpoint titers. Samples were considered seropositive when a dilution of at least 1:10 reduced the formation of viral plaques of SARS-CoV-2 by at least 90% (Algaissi and Hashem 2020; Sit et al. 2020).

### Virus Isolation

Samples that were positive by coronavirus were submitted for virus isolation in cell cultures. In brief, Vero E6 cells were cultured for 24 h in culture flasks before inoculation. The culture medium used was minimal essential medium (MEM) containing 2% fetal bovine serum and 1% antibiotic–antimycotic. The samples were inoculated onto Vero E6 cells in alternative wells of the culture flasks, as previously described (Pauvolid-Correa et al. 2022). The presence of cytopathic effect (CPE) was examined daily. For each sample, isolation was attempted in a maximum of three consecutive blind passages. Samples with no observed CPE were considered negative for cytopathic viruses.

## Results

In our study involving 72 domestic animals, all the animals were mixed breed. All the cats in the group were adults. Among the dogs, 11 were young (less than one year old), 37 were classified as adults, and five were categorized as old (over seven years old). All collected swabs from animals were negative for both SARS-CoV-2 RNA and influenza A virus, none of the tested animals had a PRNT90 titer surpassing 10, which serves as the threshold for considering a sample as positive that suggests an absence of both active SARS-CoV-2 infection and prior exposure to the virus among the studied domestic animals (Table 1).

**Table 1:** Animals Sampled and Tested for Coronavirus and Influenza A Virus at Fiocruz Atlantic Forest Biological Station and its Surroundings, and in Areas of the Pedra Branca Forest (PBF) Brazil, Rio de Janeiro, Brazil.

Animals	Ordem	Family	Species	Number of animals sampled (F/M)†	Number of animals positive for SARS-Cov-2/Influenza A	Number of animals positive for other coronaviruses (%)	SARS-CoV serology positive/tested
Domestic	Carnivora	Canidae	*Canis lupus*	53 (26/27)	0/0	NT§	0/53
		Felidae	*Felis catus*	19 (9/10)	0/0	NT	0/19
Wild	Primata	Callitrichidae	*Callithrix* sp.	66 (34/32)	0/0	0	0/58
	Didelphimorphia	Didelphidae	*Didelphis aurita*	32 (9/23)	0/0	0	0/4
			*Marmosa (Micoureus) paraguayana*	1 (1/0)	0/0	0	NT
			*Marmosops incanus*	2 (0/2)	0/0	0	NT
			*Monodelphis americana*	1 (0/1)	0/0	0	NT
	Rodentia	Cricetidae	*Akodon cursor*	1 (0/1)	0/0	0	NT
			*Oligoryzomys nigripes*	8 (3/5)	0/0	0	NT
		Muridae	*Rattus norvegicus*	3 (2/1)	0/0	0	0/1
			*Rattus rattus*	6 (5/1)	0/0	0	NT
			*Mus musculus*	2 (2/0)	0/0	0	NT
	Chiroptera	Molossidae	*Molossus molossus*	5 (5/0)	0/0	0	NT
		Vespertilionidae	*Myotis nigricans*	3 (2/1)	0/0	0	NT
			*Myotis riparius*	7 (1/6)	0/0	0	NT
		Phyllostomidae	*Anoura caudifer*	6 (3/3)	0/0	0	NT
			*Anoura geoffroyi*	5 (1/4)	0/0	0	NT
			*Artibeus cinereus*	1 (0/1)	0/0	0	NT
			*Artibeus fimbriatus*	22 (13/9)	0/0	0	NT
			*Artibeus lituratus*	118 (66/52)	0/0	6 (5,1%)	0/2
			*Artibeus obscurus*	9 (5/4)	0/0	0	NT
			*Carollia brevicauda*	7 (4/3)	0/0	1 (14,3%)	NT
			*Carollia perspicillata*	131 (61/70)	0/0	3 (2,3%)	NT
			*Chiroderma villosum*	1 (0/1)	0/0	0	NT
			*Desmodus rotundus*	12 (5/7)	0/0	1 (8,3%)	NT
			*Epitesicus brasiliensis*	1 (0/1)	0/0	0	NT
			*Glossophaga soricina*	9 (7/2)	0/0	0	NT
			*Lonchophylla peracchii*	2 (2/0)	0/0	0	NT
			*Micronycteris megalotis*	2 (0/1)	0/0	0	NT
			*Micronycteris microtis*	2 (1/1)	0/0	1 (50,0%)	NT
			*Micronycteris minuta*	1 (1/0)	0/0	0	NT
			*Phyllostomus hastatus*	2 (2/0)	0/0	1 (50%)	0/1
			*Platyrrhinus lineatus*	11 (2/9)	0/0	1 (9,1%)	NT
			*Platyrrhinus recifinus*	9 (3/6)	0/0	0	0/1
			*Sturnira lilium*	11 (7/4)	0/0	2 (18,2%)	0/1
			*Sturnira tildae*	4 (0/4)	0/0	0	NT
			*Tonatia bidens*	3 (2/1)	0/0	1 (3,3%)	NT
			*Vampyressa pusilla*	6 (2/4)	0/0	0	NT
			TOTAL	584	0/0	17	0/140

^†^F—female, M—male, § NT—not tested.

In the primate subset of our study, represented solely by the unique species hybrid *Callitrix* spp., a total of 66 animals were sampled (50 adults, 13 juveniles, and three infants). Oropharyngeal and rectal swabs from these primates were negative for tested viruses. None of the tested sera exhibited the presence of SARS-CoV-2-neutralizing antibodies (Table 1).

Among the marsupials sampled in our study, the Big-eared opossum (*Didelphis aurita* Wied-Neuwied, 1826) was the most frequently sampled species, accounting for 88.9% of the marsupial sample. The rodents in our study were from the Cricetidae and Muridae families (Table 1). All animals were classified as adults. No CoV or Influenza A viruses were detected in these marsupials and rodents. Unfortunately, only four serum samples were available for testing within this group and gave negative results (Table 1).

**Figure 3:**
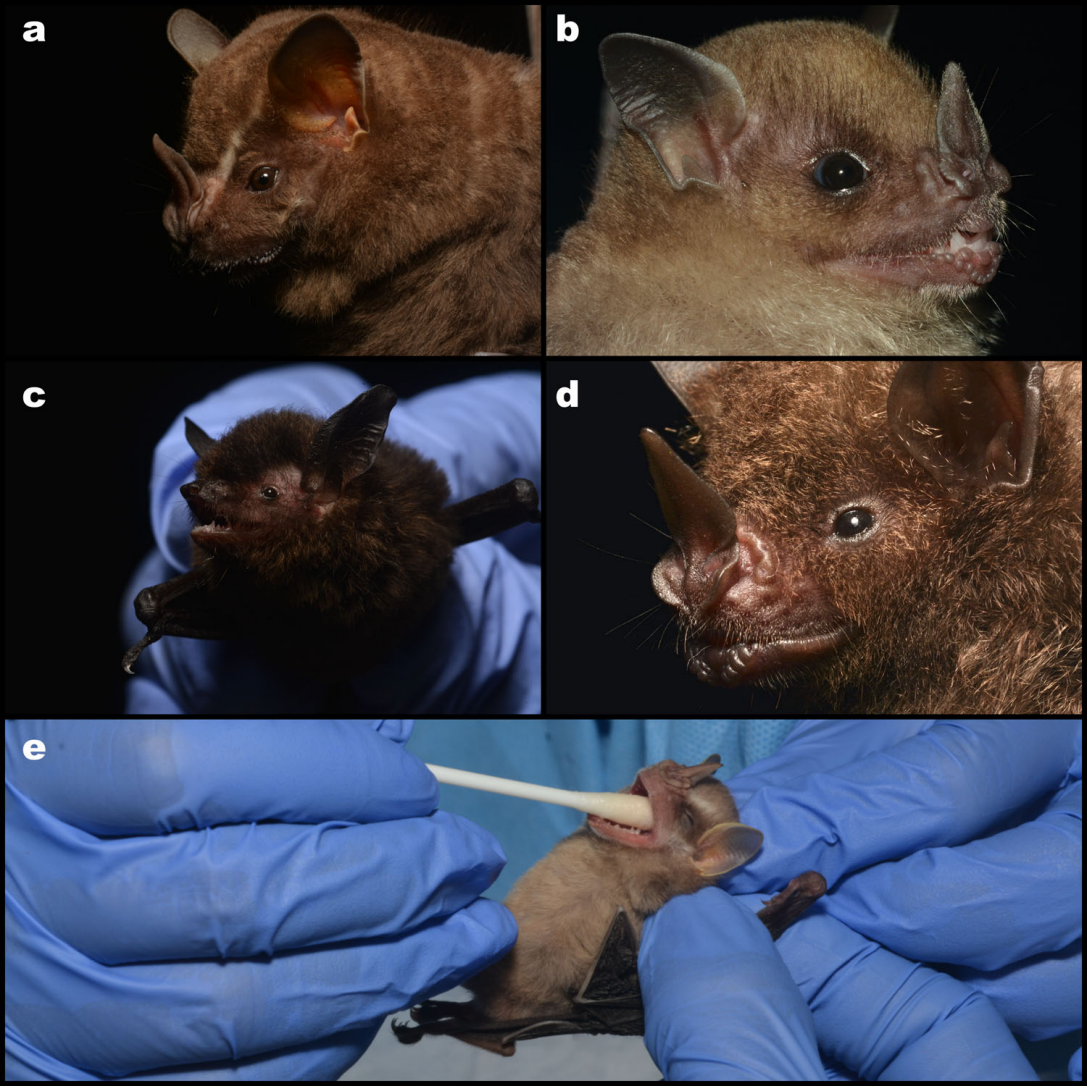
Some bats captured in the present study. A—Great fruit-eating bat (*Artibeus lituratus* (Olfers, 1818)); B—Little yellow-shouldered bat (*Sturnira lilium* (E. Geoffroy, 1810)); C—Black myotis (*Myotis nigricans* (Schinz, 1821)); D—Seba's short-tailed bat (*Carollia perspicillata* (Linnaeus, 1758)); E—oral swab collection from the Southern little yellow-eared bat (*Vampyressa pusilla* (Wagner, 1843))*,* Fiocruz Atlantic Forest Biological Station and Pedra Branca State Park, Rio de Janeiro, Brazil. Image credit: Maria Ogrzewalska.

In our study, 390 bats from 26 different species were sampled, spanning three families: Molossidae, Vespertilionidae, and Phyllostomidae (Fig. 3). Despite the variety of species tested, no SARS-CoV-2 or Influenza A viruses were detected in any of the sampled bats. No sera were available for testing for most of the bats in the study (Table 1).

*Alphacoronavirus* RNA was detected in 17 (4.3%) phyllostomid bats encompassing eight different species: *A. lituratus*, Silky short-tailed bat (*Carollia brevicauda* (Schinz, 1821)), *C. perspicillata*, Common big-eared bat (*Micronycteris microtis* Miller, 1898), Greater spear-nosed bat (*Phyllostomus hastatus* (Pallas, 1767)), White-lined broad-nosed bat (*Platyrrhinus lineatus* (E. Geoffroy, 1810)), Little yellow-shouldered bat (*Sturnira lilium* (Geoffroy 1810)), Greater round-eared bat (*Tonatia bidens* (Spix, 1823)), and Common vampire bat ((*Desmodus rotundus* (Geoffroy, 1810)) (Tables 1 and 2).

**Table 2:** Positive for Coronavirus Samples from Bats in the Present Study Captured at Fiocruz Atlantic Forest Biological Station and its Surroundings, and in Areas of the Pedra Branca Forest (PBF) Brazil, Rio de Janeiro, Brazil.

Bat ID	Sample type†	Species	Sex	Age class	Collection date	RT-PCR result	BatCoV	Virus name	GenBank
FMA 537	OS	*A. lituratus*	F	Adult	15/03/2021	Positive	Alpha	BatCoV/Artibeus_lituratus/Brazil/RJ/FIOCRUZ-A21132/2021	OR596328
	F					Negative			
FMA 605	OS	*A. lituratus*	F	Adult	26/04/2021	Positive	Alpha	Low-quality sequence	Not deposited
	RS					Negative			
FMA 520	OS	*A. lituratus*	M	Adult	09/02/2021	Negative			
	RS					Positive	Alpha	Low-quality sequence	Not deposited
FMA 563	OS	*A. lituratus*	F	Adult	22/03/2021	Negative			
	RS					Positive	Alpha	BatCoV/Artibeus_lituratus/Brazil/RJ/FIOCRUZ-A210081/2021	OR596324
FMA 566	OS	*A. lituratus*	F	Adult	22/03/2021	Negative			
	RS					Positive	Alpha	BatCoV/Artibeus_lituratus/Brazil/RJ/FIOCRUZ-A210084/2021	OR596325
FMA 533	OS	*A. lituratus*	F	Adult	15/03/2021	Negative			
	RS					Positive	Alpha	BatCoV/Artibeus_lituratus/Brazil/RJ/FIOCRUZ-A210104/2021	OR596326
FMA 573	OS	*C. brevicauda*	M	Adult	22/03/2021	Positive	Alpha	BatCoV/Carollia_brevicauda/Brazil/RJ/FIOCRUZ-A210067/2021	OR596323
	RS					Not collected			
FMA 560	OS	*C. perspicillata*	M	Adult	22/03/2021	Positive	Alpha	Low-quality sequence	Not deposited
	RS					Negative			
FMA 536	OS	*C. perspicillata*	F	Juvenile	15/03/2021	Negative			
	RS					Positive	Alpha	BatCoV/Carollia_perspicillata/Brazil/RJ/FIOCRUZ-A210106/2021	OR596327
FMA 611	OS	*C. perspicillata*	F	Adult	26/04/2021	Negative			
	RS					Positive	Alpha	Low-quality sequence	Not deposited
FMA 1004	OS	*D. rotundus*	M	Adult	05/07/2022	Negative			
	F					Positive	Alpha	Low-quality sequence	Not deposited
FMA 604	OS	*M. microtis*	F	Adult	26/04/2021	Positive	Alpha	Low-quality sequence	Not deposited
	RS					Not collected			
FMA 598	OS	*P. hastatus*	F	Adult	26/04/2021	Negative			
	RS					Positive	Alpha	BatCoV/Phyllostomus_hastatus/Brazil/RJ/FIOCRUZ-A21309/2021	OR596329
FMA 1015	OS	*P. lineatus*	F	Adult	05/07/2022	Negative			
	F					Positive	Alpha	Low-quality sequence	Not deposited
FMA 601	OS	*S. lilium*	F	Adult	26/04/2021	Negative			
	F					Positive	Alpha	BatCoV/Sturnira_lilium/Brazil/RJ/FIOCRUZ-A21315/2021	OR596330
FMA 664	OS	*S. lilium*	M	Adult	14/06/2021	Negative			
	RS					Positive	Alpha	Low-quality sequence	Not deposited
FMA 608	OS	*T. bidens*	F	Adult	26/04/2021	Positive	Alpha	Low-quality sequence	Not deposited
	F					Negative			

^†^OS—Oral swab, RS—rectal swab, F—faces.

**Fig. 4:**
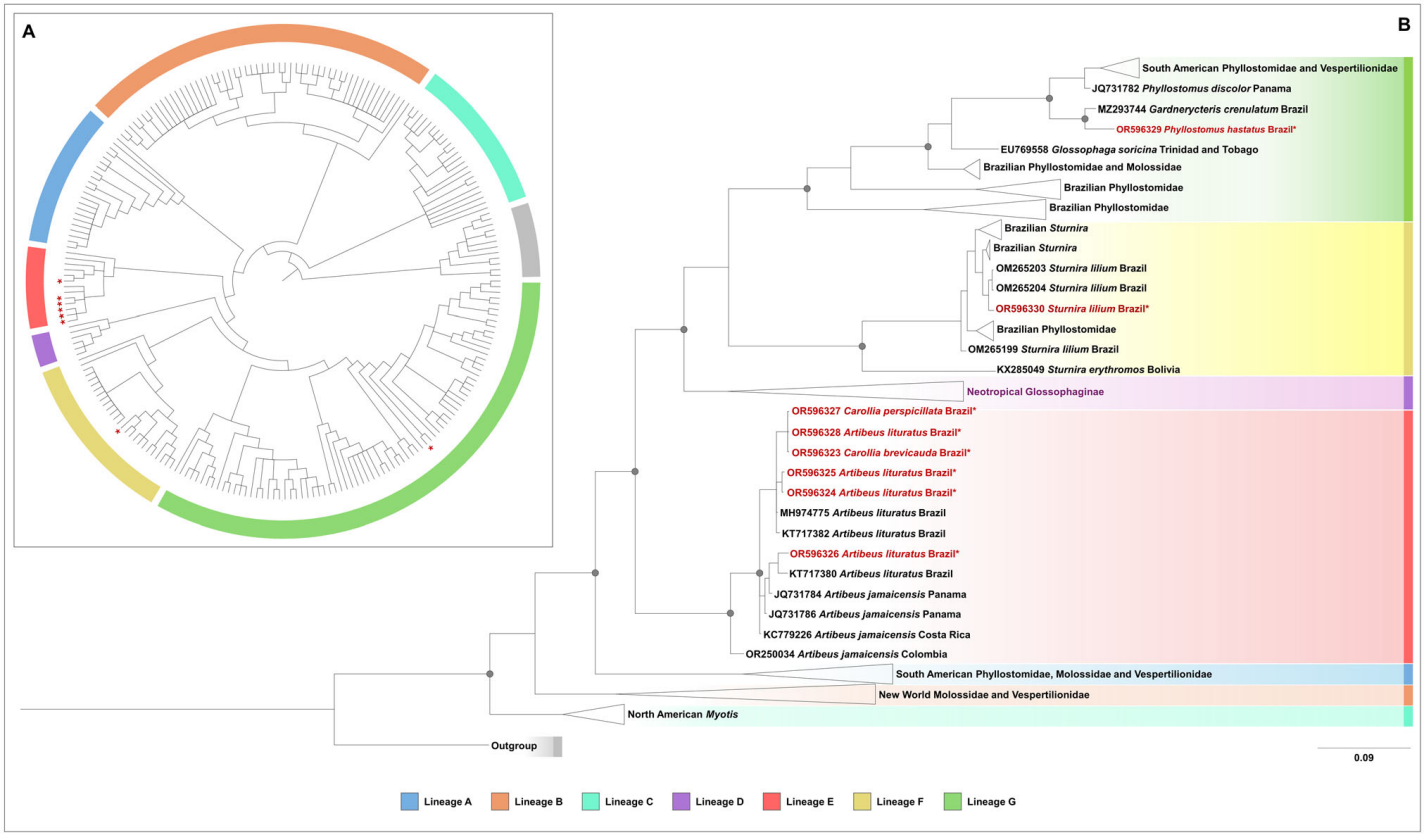
Phylogenetic relationships of *Alphacoronavirus* from New World bats based on Bayesian Inference using the *RdRp* partial gene. The colored lineages follow Caraballo et al. (2022). Samples from the Fiocruz Atlantic Forest Biological Station and Pedra Branca State Park, Rio de Janeiro, Brazil, are highlighted in red

High-quality consensus sequences (> 400 bp) were successfully obtained from only eight samples and deposited in GenBank with assigned numbers detailed in Table 2. The sequences displayed a similarity range of 90.4% to 99.3% with other coronavirus sequences derived from South American bats. Notably, all viruses obtained from *A. lituratus*, *C. brevicauda*, and *C. perspicillata* grouped in Lineage E, while one virus obtained from *S. lilium* clustered in Lineage F, and another from *P. hastatus* fell into Lineage G (Fig. 4, Fig. 1 Supp). We could observe that the BatCoV sequences grouped according to the main bat hosts, that is, sequences from the same bat species or genus were virtually grouped in monophyletic branches.

Despite these successful sequence analyses, it was not feasible to isolate the virus from any of the RT-PCR positive samples.

## Discussion

SARS-CoV-2 has been reported in animals across 36 countries affecting animals from various orders (WOAH 2023). Indeed, there have been reports of SARS-CoV-2 infections in pets worldwide, including South America (Calvet et al. 2021; Carlos et al. 2021; Fuentealba et al. 2021; Schiaffino et al. 2021; Agopian et al. 2022). Despite these studies, it is widely acknowledged that cats and dogs do not play a significant role in the epidemiology of SARS-CoV-2 and several studies have found no evidence of infection or exposure in pets (Patterson et al. 2020; Temmam et al. 2020; Dias et al. 2021; Sanchez-Montes et al. 2022). The absence of evidence of animal exposure to the virus in our study may also be attributed to the fact that the sampled animals predominantly reside outside homes, minimizing close contact with humans.

The analysis of the angiotensin-converting enzyme 2 (ACE2) receptor across diverse vertebrates indeed suggests a potentially wide range of SARS-CoV-2–susceptible mammal host species, including many South American species (Damas et al. 2020). However, there are currently limited data available on the exposure and infection of South American wildlife to SARS-CoV-2 in their natural habitats (de Abreu et al. 2021; Sacchetto et al. 2021; Stoffella-Dutra et al. 2023). Our study aimed to broaden the understanding of SARS-CoV-2 exposure in South American wildlife, bats, primates, rodents, and marsupials. Despite our efforts, we did not obtain any positive results for SARS-CoV-2 in these animals.

Marmosets *Callithrix* have adapted to synanthropic environments, and in Brazil, they are sometimes kept as pets and are frequently fed by people in urban parks (Fig. 2A). This close coexistence poses a substantial potential source of infection. Despite the experimental evidence of susceptibility of marmoset to SARS-CoV-2 infection (Lu et al. 2020), there is limited understanding regarding the potential for viral infection in natural settings. However, our study's results align with an earlier investigation exploring the potential natural infection of free-living neotropical nonhuman primates (NHPs) with SARS-CoV-2 (de Abreu et al. 2021; Sacchetto et al. 2021) where no evidence of SARS-CoV-2 infection was found. Nonetheless, in one study, natural SARS-CoV-2 infection in the black-tailed marmoset (*Mico melanurus* (Geoffroy, 1812) from an urban area in Mid-West Brazil was reported (Pereira et al. 2022) emphasizing the further surveillance in free-ranging NHP.

Several rodent species are predicted to be potential hosts of SARS-CoV-2 and may also harbor other coronaviruses, creating the potential for viral recombination, including recombination of SARS-CoV-2 with other coronaviruses (Miot et al. 2022; Fisher et al. 2023; Wang et al. 2023). Our study's results align with findings from studies in Europe and Asia that did not detect SARS-CoV-2 infection in tested rats and rodents (Colombo et al. 2022; Miot et al. 2022; Wernike et al. 2022; Fisher et al. 2023). However, these results should be interpreted with caution due to the limited number of animals tested. A similar situation was observed in marsupials, where neither active infection was detected by RT-PCR nor antibodies indicating past infection were found in the tested sera. Although these initial findings are constrained by the small sample size, they provide valuable new insights into the potential susceptibility of marsupials to SARS-CoV-2.

Bats harbor a diverse array of coronaviruses, some of which are related to viruses causing human respiratory syndromes such as MERS, SARS, and COVID-19 (Calisher et al. 2006; Moratelli and Calisher 2015); however, the absence of SARS-CoV-2 in the tested bats aligns with expectations. Coronaviruses in bats are influenced by both species’ richness and geographical distribution, demonstrating clustering at the bat genus level. These genus-specific clusters are often associated with distinct CoVs species (Leopardi et al. 2018; Fan et al. 2019) and SARS-related CoVs are found primarily within the Old-World bat families. Moreover, experimental studies involving Neotropical bat species, the Big brown bat (*Eptesicus fuscus* (Beauvois, 1796)) (Hall et al. 2021) and the Brazilian free-tailed bat (*Tadarida brasiliensis* (Geoffroy, 1824), Molossidae) (Bosco-Lauth et al. 2022), indicate that these species are minimally competent hosts for SARS-CoV-2.

Our observations of CoVs in bats align with the existing understanding of coronavirus diversity in Brazilian bats where mainly *Alfacoronavirus* have been identified in over 20 bat species spanning the Phyllostomidae, Molossidae, and Vespertilionidae families with the prevalence ranging from 0–17.2% (Brandao et al. 2008; Corman et al. 2013; Hernández-Aguilar et al. 2021; Lima et al. 2013; Moreira-Soto et al. 2015; Simas et al. 2015; Asano et al. 2016; Goes et al. 2016; Bittar et al. 2020; Alves et al. 2021; Bueno et al. 2022; Violet-Lozano et al. 2023) and our findings agree with previous studies that CoVs exhibit genus specificity rather than species specificity (Carrington et al. 2008; Anthony et al. 2013; Corman et al. 2013).

Our finding that viruses from *Artibeus* and *Carollia* clustered in the same clade suggests an interesting ecological host factor at play, potentially related to their feeding behavior. The fact that these two bat genera may visit the same fruits could facilitate a hypothetical exchange of viruses between them and sporadic observations of closely related coronaviruses in different bat species and even families have been reported previously (Anthony et al. 2013; Bittar et al. 2020; Bueno et al. 2022). These findings highlight the dynamic nature of virus transmission among bats, and ecological factors, particularly those related to bat behavior and habitat, can play a significant role in shaping the patterns of virus distribution and clustering.

As VERO E6 cells are successfully used for SARS-CoV-2 virus isolation in our laboratory, we tried unsuccessfully to isolate *Alphacoronavirus* from bats using the same technique. The unsuccessful attempt at virus isolation, with no cytopathic effect (CPE) observed in any of the Vero cell lines, raises several possibilities. It is possible that Vero cells are not susceptible to infection by the specific BatCoVs present in these samples (Bonny et al. 2017) or our samples may contain noninfectious virus particles. This could happen if the virus is damaged or if the viral load in the sample is too low to establish an infection in the cells and the quantity of virus in the sample might also be too low to establish a productive infection in the cell culture.

The suggestion that new SARS-CoV-2 variants may acquire substitutions at the receptor-binding domain of the spike protein, potentially facilitating infectivity in mice and/or rats compared to the original pandemic strain, is an important consideration (Shuai et al. 2021). While our present study did not find evidence of SARS-CoV-2 exposure in either domestic or wild animals, the emphasis on the importance of monitoring and understanding the potential for SARS-CoV-2 transmission between humans and animals is well founded. Surveillance of such cases is crucial for gaining a better understanding of how animals exposed to COVID-19 might be affected, identifying potential hosts, and continually monitoring for any changes in the virus in animals. These efforts are essential for comprehending how the virus evolves, the emergence of new variants, and the potential threats they might pose to public health, as outlined by the World Organization for Animal Health in 2022.

In the present study, we did not detect Influenza A viruses. Influenza A viruses have been detected in a variety of mammals, as well as in many different aquatic birds, considered the natural reservoirs for avian influenza viruses (AIVs) (Webster et al. 1992). Currently, H17N10 and H18N11 subtypes have been described in fruit bats in Guatemala, Peru (Tong et al. 2012, 2013) and Brazil (Campos et al. 2019). These recent findings indicate that bats constitute a potentially important and likely ancient reservoir for a diverse pool of Influenza A viruses; however, these phylogenetically highly divergent avian-associated Influenza A viruses in their hemagglutinin (HA) and neuraminidase (NA) genes have only been found so far in six individual bat specimens. The absence of this virus in our study may be related to the very low (0–1%) prevalence of the virus in neotropical bats (Tong et al. 2012, 2013; Campos et al. 2019; Violet-Lozano et al. 2023).

## Conclusions

Our study makes a significant contribution to our understanding of coronavirus diversity in neotropical bats, emphasizing the importance of ongoing research in this field. The findings underscore several key points, such as (1) the association of CoVs with various bat host genera, which contributes to our understanding of the complex interactions between coronaviruses and their natural hosts, (2) potential threat for the emergence of novel CoVs: the observation that certain CoVs can infect bats across diverse genera suggests the potential for the ongoing evolution of CoVs in bats, posing a potential threat for the emergence of novel coronaviruses in new hosts, (3) need for continued monitoring: our study supports the need for the continued monitoring of wild species as part of ongoing SARS-CoV-2 surveillance. This proactive approach is essential for early detection, surveillance, and understanding the dynamics of coronaviruses in wildlife, helping to mitigate potential risks of spillover events.

## Conflict of interest

The authors have declared that no competing interests exist.

## Supplementary Information

Below is the link to the electronic supplementary material.

Supplementary file1 (DOCX 17 KB)

Supplementary file2 (XLSX 110 KB)

Supplementary file3 (TIFF 6472 KB)

## Data Availability

Data that support the findings of this study are available from the corresponding author upon reasonable request.
