# Supplementary material for: Cross-Species Surveillance of Respiratory Viruses in Domestic and Wild Mammals of an Urban Atlantic Forest from Brazil
Source: Ecohealth. 2025 Feb 4;22(1):11–28. doi: 10.1007/s10393-024-01691-w (PMC11890330; doi:10.1007/s10393-024-01691-w)
Supplement: Supplementary file 1 — Supplementary file1 (DOCX 17 KB) [file 10393_2024_1691_MOESM1_ESM.docx]

**Supplementary Table 1.** Sequences of Alphacoronavirus used for phylogenetic analyses from bats at Fiocruz Atlantic Forest Biological Station and Pedra Branca State Park, Rio de Janeiro, Brazil

**Figure 1. Supplementary.**Phylogenetic relationships of *Alphacoronavirus* from New World bats based on Bayesian Inference using the *RdRp* partial gene. The colored lineages follow Caraballo et al. (2022). Samples from the Fiocruz Atlantic Forest Biological Station and Pedra Branca State Park, Rio de Janeiro, Brazil are highlighted in red.
