# Supplementary figures and images for: Cross-Species Surveillance of Respiratory Viruses in Domestic and Wild Mammals of an Urban Atlantic Forest from Brazil
Source: Ecohealth. 2025 Feb 4;22(1):11–28. doi: 10.1007/s10393-024-01691-w (PMC11890330; doi:10.1007/s10393-024-01691-w)

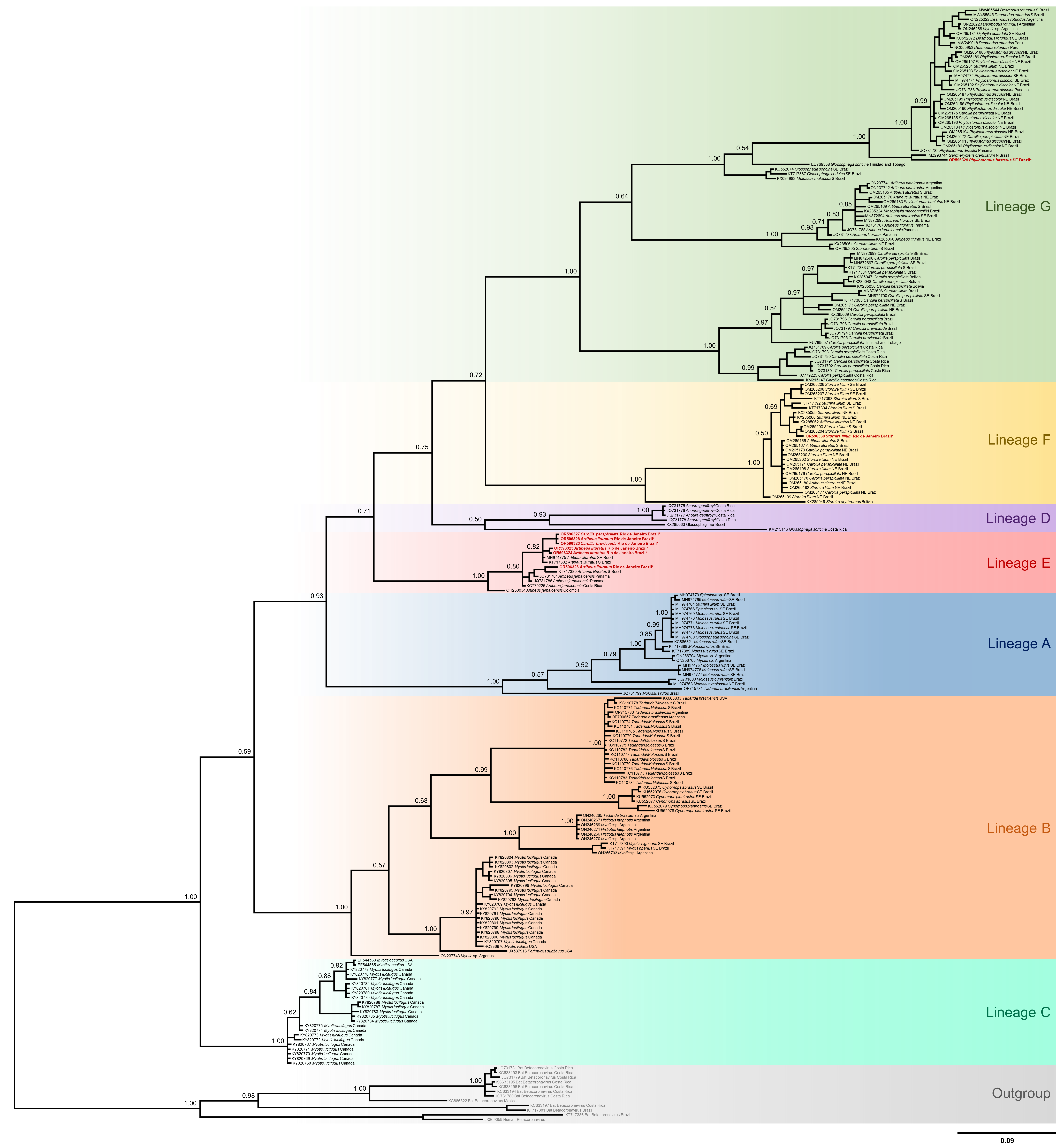

Supplement: Supplementary file 3 — Supplementary file3 (TIFF 6472 KB) [file 10393_2024_1691_MOESM3_ESM.tiff]
